# Can optical flow perturbations detect walking balance impairment in people with multiple sclerosis?

**DOI:** 10.1371/journal.pone.0230202

**Published:** 2020-03-10

**Authors:** Brian P. Selgrade, Diane Meyer, Jacob J. Sosnoff, Jason R. Franz

**Affiliations:** 1 Joint Department of Biomedical Engineering, University of North Carolina at Chapel Hill and North Carolina State University, Chapel Hill, NC, United States of America; 2 UNC Healthcare, UNC Center for Rehabilitation Care, Chapel Hill, NC, United States of America; 3 Department of Kinesiology and Community Health, University of Illinois at Urbana-Champaign, IL, United States of America; Tokai University, JAPAN

## Abstract

People with multiple sclerosis (PwMS) who exhibit minimal to no disability are still over twice as likely to fall as the general population and many of these falls occur during walking. There is a need for more effective ways to detect preclinical walking balance deficits in PwMS. Therefore, the purpose of this study was to investigate the effects of optical flow perturbations applied using virtual reality on walking balance in PwMS compared to age-matched controls. We hypothesized that susceptibility to perturbations–especially those in the mediolateral direction–would be larger in PwMS compared to controls. Fourteen PwMS and fourteen age-matched controls walked on a treadmill while viewing a virtual hallway with and without optical flow perturbations in the mediolateral or anterior-posterior directions. We quantified foot placement kinematics, gait variability, lateral margin of stability and, in a separate session, performance on the standing sensory organization test (SOT). We found only modest differences between groups during normal, unperturbed walking. These differences were larger and more pervasive in the presence of mediolateral perturbations, evidenced by higher variability in step width, sacrum position, and margin of stability at heel-strike in PwMS than controls. PwMS also performed worse than controls on the SOT, and there was a modest correlation between step width variability during perturbed gait and SOT visual score. In conclusion, mediolateral optical flow perturbations revealed differences in walking balance in PwMS that went undetected during normal, unperturbed walking. Targeting this difference may be a promising approach to more effectively detect preclinical walking balance deficits in PwMS.

## Introduction

Multiple sclerosis (MS) affects over 2.3 million people, and its prevalence is increasing [1]. Most people with MS (PwMS) have relapsing-remitting MS, and peak incidence occurs around age 30. Accordingly, MS is the most common neurological disease among middle-aged adults, presenting significant and enduring rehabilitative challenges to preserve mobility. Symptoms of MS arise from demyelination around axons and include impaired proprioceptive acuity, which in turn leads to poor control of balance [2–4]. Furthermore, up to 86% of patients with relapsing-remitting MS have vestibulopathies [5], which likely impair their balance [6]. Indeed, an estimated 56% of PwMS fall annually, and 37% of PwMS are frequent fallers [7]. Compounding this exceptionally high risk of falls, PwMS have deficiencies in bone density and vitamin D [8], which makes them nearly 3 times more likely than the general population to suffer hip fracture [9].

Given the severe personal and economic consequences of falls, there is a need for effective approaches to detect balance impairment in PwMS to empower the timely and discriminate prescription of interventions. However, pre-clinical balance impairment prior to a first fall may go undetected using conventional diagnostic efforts. Even PwMS who exhibit minimal to no disability on the Expanded Disability Status Scale are still over twice as likely to fall as the general population [7]. However, most falls occur during dynamic activities like walking [7, 10, 11], yet clinical tests disproportionately focus on standing balance integrity.

In contrast to standing balance, walking balance is complex and highly dynamic. Nevertheless, several key outcome measures have emerged to quantify fundamental characteristics considered relevant to walking balance integrity. Foremost, balance disturbances during walking are accommodated by systematic changes to foot placement (*i*.*e*., step width and step length). Changing foot placement to preserve walking balance is indicative of underlying corrective motor responses that, in the presence of balance disturbances, manifest as higher step-to-step variability. Accordingly, step width variability (SWV) and step length variability (SLV) are generally considered markers of reduced walking balance integrity. For example, PwMS demonstrate higher SWV and SLV than age-matched controls [12, 13]. However, changes in foot placement must also be meaningfully orchestrated to ensure that the body’s center of mass (CoM) remains within the base of support, in order to prevent a fall. Indeed, larger corrective motor responses via foot placement are often necessitated by larger disturbances in trunk sway and related movement of the body’s CoM. Margin of stability (MoS) is an outcome that relates position and velocity of the body’s CoM to the safety margins prescribed by the base of support (i.e., the lateral boundary of the feet). Here, larger positive values of MoS (*i*.*e*., a larger safety margin between the CoM and the lateral edge of the stance foot) and/or lower MoS variability are interpreted to suggest higher walking balance integrity. In PwMS with clinical gait impairment, for example, MoS was smaller than in healthy controls, and mediolateral MoS correlated with number of falls [14]. Thus, margin of stability, its determinants (i.e. foot placement and CoM position), and their respective variabilities provide a reasonably robust combination of metrics to understand the onset and progression of walking balance impairment in PwMS.

To maximize our ability to detect subtle impairments, it may be necessary to challenge underlying impairments in sensorimotor control caused by MS. Recent evidence suggests that PwMS are more reliant on visual feedback for motor planning and execution than the general population [15]. Accordingly, we posit that PwMS may be more susceptible to the presence of optical flow balance perturbations applied during walking in virtual reality. Optical flow perturbations evoke the perception of instability, and their presence during walking elicits increases in gait variability that scale with perturbation amplitude [16, 17]. These effects are also direction-dependent—walking balance, at least in young adults, is much more susceptible to mediolateral (ML) than anterior-posterior (AP) optical flow perturbations [18, 19]. This is not surprising, as lateral balance in walking depends more on sensory integration than anterior-posterior balance, which benefits from passive stability [20, 21]. Moreover, promising outcomes from earlier studies have shown that, compared to young adults, optical flow perturbations induce larger decreases in metrics of walking balance integrity in older adults, with more pronounced effects in those with a history of falls [22, 23]. These results are highly relevant to the detection of preclinical balance impairment in PwMS; older adults also tend to rely more than young adults on vision for movement control. This reliance on vision is thought to compensate for disproportionate proprioceptive, somatosensory or vestibular declines and associated problems with sensory re-weighting [24–26].

Therefore, the purpose of this pilot study was to investigate the effects of optical flow perturbations on gait variability and lateral margin of stability during walking in PwMS compared to age-matched controls. We first hypothesized that susceptibility to optical flow perturbations would be larger in people with MS compared to age-matched controls. We also hypothesized that this susceptibility would be larger for ML perturbations than AP perturbations. To better understand underlying changes in sensorimotor coordination in PwMS that may be associated with responses to optical flow perturbations, we also used the sensory organization test (SOT), which measures the ability to use individual senses for balance control, to find correlates between susceptibility to perturbation and deficits in using specific senses to maintain balance [27, 6]. This research will elucidate the potential of optical flow perturbations for identifying balance deficits in PwMS.

## Methods

### Study participants

Sixteen people with relapsing-remitting MS and sixteen healthy, age-matched controls participated in this study. All participants provided written, informed consent before participating. All experimental procedures were approved by the University of North Carolina Biomedical Sciences Institutional Review Board (IRB number 17–2281 and FWA number 4801). Participants were excluded if they required over 6 seconds to complete a 25-foot (i.e., 7.62 m) walk test, had spasticity that was not well-controlled by medication, had a relapse of MS within the 30 days prior to testing, or were over 55 years of age. Although MS is more prevalent in older individuals [28], this study was motivated by the need to detect preclinical balance impairment, so we designed these exclusion criteria to test younger PwMS with milder impairment. One PwMS and her control were excluded from the final analysis because she was unable to complete the treadmill walking trials without holding onto the handrail. We excluded a second pair of participants from our analysis because the control participant’s primary outcomes fell >5 standard deviations from the mean calculated from the remaining subjects. Thus, we report data for 14 PwMS (average ± standard deviation, age: 38.9±6.6 years, body mass: 81.2±18.7 kg, height: 1.7±0.1 m, 12 female) and 14 age-matched controls (age: 38.2±6.9 years, body mass: 76.2±17.4 kg, height: 1.7±0.1 m).

### Experimental protocol and instrumentation

Participants completed the study over the course of 2 separate visits: a session completed in a rehabilitation clinic setting and a session completed in the lab. These visits typically occurred within 1–2 weeks of each other. In the rehabilitation clinic, a licensed physical therapist administered the NeuroCom^®^ SOT [27]. The NeuroCom^®^ system consists of a force platform and 3 walls with a scene spanning the user’s visual field. The SOT estimates CoM movement across 6 different conditions to isolate how well individuals use individual sensory modalities (*i*.*e*., visual, vestibular, and somatosensory) to maintain standing balance. According to standardized procedures, each subject completed three, 20-second trials for each of the six conditions from which we derived the following outcome measures: the visual score (SOT-Vi), vestibular score (SOT-Ve), somatosensory score (SOT-S) and composite score (SOT-C). The SOT has high test-retest reliability in PwMS [6] and can differentiate between elderly people with and without balance dysfunction based on history of falls [29].

In order to consider our results relative to a common clinical balance assessment, a licensed physical therapist also administered the Berg Balance Scale (BBS) to each study participant [30]. This assessment involves 14 functional tests (e.g. turning, standing on one leg) with a maximum score of 56. Low scores indicate poor balance. Prior studies of PwMS show that those with a history of falls score significantly lower on the BBS and that BBS scores correlate with other assessments such as the dynamic gait index [31].

During the laboratory session, participants first completed the modified fatigue impact scale (MFIS) [32] and activity-specific balance confidence scale (ABC) [33]. Next, a Semmes-Weinstein monofilament test with a 4-2-1 design performed on the plantar surface of the foot determined tactile sensitivity on the heel pad and under the fifth metatarsal head. Participants also performed two standardized walking tests: (*i*) a 25-foot walk test [34] completed 3 times to determine their maximum, safe walking speed, and (*ii*) a 10 m walking test to determine their preferred walking speed (PWS) from the average of three times taken to traverse an instrumented overground walkway. The latter of these tests also determined the initial speed for subsequent treadmill walking trials. We placed retroreflective markers bilaterally on the participants’ first and fifth metatarsal heads, calcanei, lateral ankles, knee, anterior superior iliac spine, posterior superior iliac spine, sacrum and seventh cervical vertebra. We also placed marker clusters bilaterally on the shank and thigh. Participants walked on an instrumented treadmill (Bertec, Columbus, OH, USA) for at least 5 minutes at their preferred overground walking speed to acclimate to treadmill walking and allow their movement patterns to stabilize. If a participant indicated that the preferred overground speed was uncomfortably after a few minutes, we reduced the treadmill speed by 10% for the experimental walking trials.

After acclimating to the treadmill, participants completed a series of 3-minute walking trials in randomized order. For each, subjects viewed a virtual hallway that was rear-projected onto a semi-circular, curved screen positioned in front of the treadmill [17, 35, 36]. The optical flow of the hallway in the direction of motion was prescribed to match the speed of the treadmill. Participants completed one walking trial with this baseline, “normal” optical flow (i.e. constant speed optical flow with no perturbations). In other trials, we applied to this baseline motion continuous optical flow perturbations to the foreground of the virtual hallway. To ensure that the visual perception of self-motion induced by these perturbations was complex and difficult to anticipate, they were created as the sum of 3 sine waves: one with an amplitude of 35 cm applied at 0.25Hz and two with amplitudes of 17.5 cm applied at 0.125 Hz and 0.442 Hz [17, 35, 36]. In one trial, optical flow was perturbed in the AP direction, thereby eliciting the visual perception that the hallway was accelerating and decelerating at random. In another trial, optical flow was perturbed in the ML direction, thereby eliciting the visual perception of lateral instability. Finally, to account for any differences in preferred walking speed between PwMS and their age-matched controls, we invited control participants to complete one additional trial with normal optical flow at the preferred speed of their associated PwMS (“matched speed”). All participants had over 2 minutes of rest between each treadmill walking condition. During each condition, we collected three-dimensional marker position data at 100 Hz using 9 cameras from a 14-camera motion analysis system (Motion Analysis Corp., Santa Rosa, CA).

### Data analysis

We filtered marker trajectories using a fourth-order, low-pass, Butterworth filter with a 16-Hz cutoff frequency. We focused our analysis on the first minute of each condition to quantify between-group differences in the initial response to perturbations compared to normal walking. Motor adaptation that may have occurred over the span of each 3-minute perturbation condition were outside the scope of the present paper. We found the instant of each heel-strike and toe-off from the times when the heel marker was at a maximum distance anterior or posterior to the sacrum, respectively [37]. Using these gait events, we determined time series of step widths (SW) by averaging heel marker positions during midstance prior to heel rise (i.e., 12–25% of the gait cycle) and finding the ML distance between consecutive steps [38]. We calculated time series of step lengths (SL) by adding treadmill translation distance during each step to the AP distance between heel markers at 20% of successive gait cycles. From these time series, we calculated subject average SL and SW for each condition. We calculated standard deviations of step length and step width to determine SLV and SWV, respectively. Similarly, we calculated standard deviations of ML motion of the sacrum, a surrogate for CoM motion, and of the seventh cervical vertebra (C7), a surrogate for trunk motion. Finally, we calculated trunk sway variability as the standard deviation of the instantaneous difference between ML C7 and sacrum position.

To gain additional insight into between-group and perturbation effects on the relation between CoM motion and foot placement, we used published procedures to calculate lateral margin of stability (MoS) throughout each stride. MoS estimates the distance between one’s extrapolated CoM (XCoM) and base of support defined by the stance foot [39, 40]. Specifically, based on previously published methods [40, 41], we calculated XCoM using Eq 1,

$$XCoM=X+V_{CoM}/\omega_{0}\;\mathrm{where}\;\omega_{0}=\sqrt{g/L}$$ (1)

where V_CoM_ is center of mass velocity, X is sacrum position, g is acceleration due to gravity (9.81 m/s^2^) and L is average leg length measured from the heel marker to the sacrum marker. We defined the lateral base of support (BoS) as the instantaneous ML position of the fifth metatarsal head during stance [42] and subtracted it from the lateral XCoM coordinate to calculate MoS using Eq 2.

MoS=BoS–XCoM (2)

Our sign convention was such that MoS would be positive if XCoM was medial to the BoS. For each stride, we found the minimum stance phase MoS and MoS at heel-strike. Additionally, we calculated the variability of minimum MoS and MoS at heel-strike using the standard deviations of their respective time series.

### Statistics

We analyzed step width, step length, gait variability, and MoS from the treadmill walking trials using a mixed model, 2-way (condition × group) ANOVA in which condition served as a within subject factor using an alpha level of 0.05. When a significant main effect or interaction was found, we focused our post hoc, pairwise comparisons as follows. First, paired-samples t-tests compared each of the two perturbation conditions to values obtained during unperturbed walking. Second, independent-samples t-tests compared PwMS and their age-matched controls at each condition. For all variables, Shapiro-Wilk tests determined if data were normally distributed. Only the results of the SOT were not normally distributed, so we instead used Wilcoxon-Mann-Whitney tests for SOT outcomes. We report effect sizes for ANOVA results using partial eta square ($\eta_{p}^{2}$) and for pairwise comparisons using Cohen’s d. To determine if treadmill speed affected each variable, paired t-tests assessed differences in control subjects between unperturbed walking at their PWS and at the preferred speed of their corresponding PwMS.

Subsequently, we conducted post-hoc Pearson correlation analyses to find relationships between SOT sores and gait variability. We focused on SWV, because it has a consistently strong response to optical flow perturbations and relates to risk of falling and disability status [43, 44]. Both controls and PwMS were included in these analyses in order to determine correlations over the widest possible ranges of SWV and SOT scores.

## Results

### Clinical outcomes and subject demographics

PwMS and control subjects did not significantly differ in age (p = 0.78), body mass (p = 0.47), or height (p = 0.31). PwMS walked 5.2% slower at preferred speed (1.29±0.11 vs. 1.36±0.11 m/s, p = 0.030, d = 0.71), 12.5% slower at fast speeds (3.76±0.47 vs. 3.29±0.38 s on the 25’ walk test, p = 0.007, d = 1.11), and reported more total fatigue (p = 0.016, d = 0.98) and cognitive fatigue (p = 0.015, d = 1.13) than their controls. PwMS also had significantly lower SOT composite, visual, and vestibular scores (p = 0.007, d = 1.15; p = 0.019, d = 0.99; and p = 0.008, d = 1.11, respectively) (Table 1), although their average composite score was similar to normative levels (69.1 vs. 70.0). Both groups had similarly high scores on the BBS (55.2±1.93 for PwMS versus 55.9±0.27 for controls; p = 0.068), as only 1 control subject and 3 PwMS scored below the maximum score of 56. Thirteen PwMS and their controls completed the ABC scale, which revealed no significant differences in balance confidence between groups (90.4±8.3 for PwMS versus 93.8±7.2 for controls; p = 0.280). Similarly, there were no significant differences between PwMS and controls in height, weight, nor plantar sensitivity. 3 PwMS in the study reported falling in the year prior to the study. These subjects tended to be older than the group average age (all > 40 years), but did not notably differ in their clinical or laboratory-based outcome measures.

**Table 1:** Clinical test results for people with MS and age-matched controls.

	SOT-C	SOT-Vi	SOT-Ve	SOT-S	BBS	SWM-heel	SWM-MT1	MFI- total	MFI- cognitive	MFI-physical
PwMS	**69.1±11.2**	**71.6±20.8**	**53.7±18.6**	96.6±2.3	55.2±1.93	4.3±0.52	3.8±0.53	**26.1±15.0**	**14.5±7.7**	10±7.7
control	**79.3±5.7**	**87.1±7.7**	**70.4±10.3**	97.9±1.5	55.9±0.27	4.1±0.26	3.6±0.28	**12.4±13.0**	**6.4±6.5**	5.1±6.4

Results are average ± standard deviation. Significant differences between PwMS and controls are in bold. Abbreviations: PwMS–people with multiple sclerosis; SOT–sensory organization test (C-composite score, Vi-visual sub-score, Ve-vestibular sub-score, S-somatosensory sub-score), BBS–Berg Balance Scale, SWM–Semmes-Weinstein monofilament threshold, MT1 –first metatarsal headMFI–modified fatigue impact scale.

### Between-group differences during unperturbed walking

**Fig 1:**
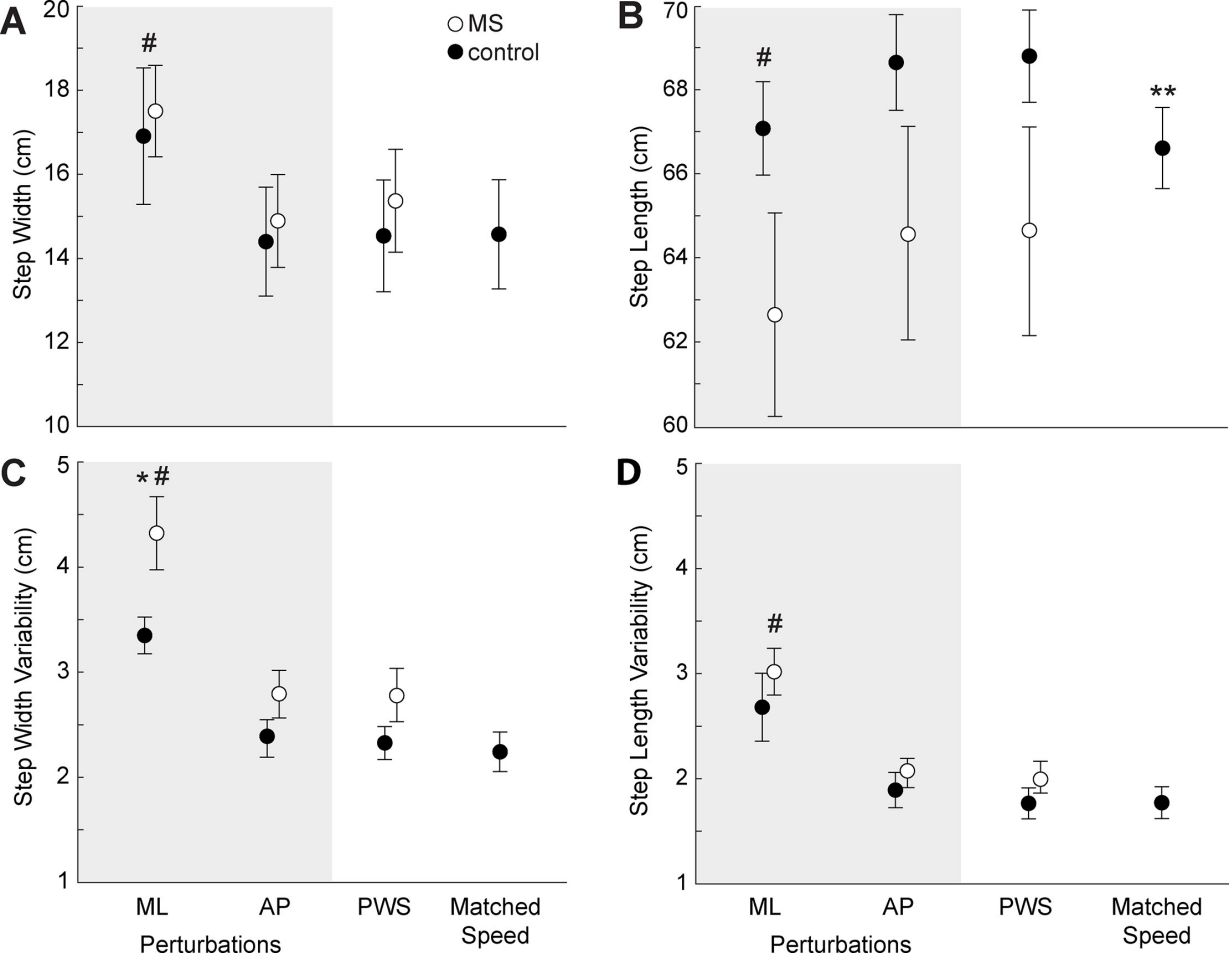
Step kinematics in PwMS versus age-matched controls across walking tasks. Group average (standard error) step width (Panel A), average step length (Panel B), step width variability (Panel C), and step length variability (Panel D) for people with multiple sclerosis (PwMS) and age-matched controls over 1 minute of walking per condition. Conditions include mediolateral (ML) and anterioposterior (AP) optical flow perturbations and two control trials. The PWS and matched speed trials have unperturbed optical flow at the participant’s preferred walking speed and the preferred walking speed of the matched PwMS, respectively. Single asterisks (*) denote significant differences (p<0.05) between PwMS and controls for a given condition. Pound signs (#) denote significant effects (p<0.05) of perturbations compared to unperturbed walking (PWS) across both groups. ^ denotes a significant difference between PWS and matched speed conditions in control participants only.

**Fig 2:**
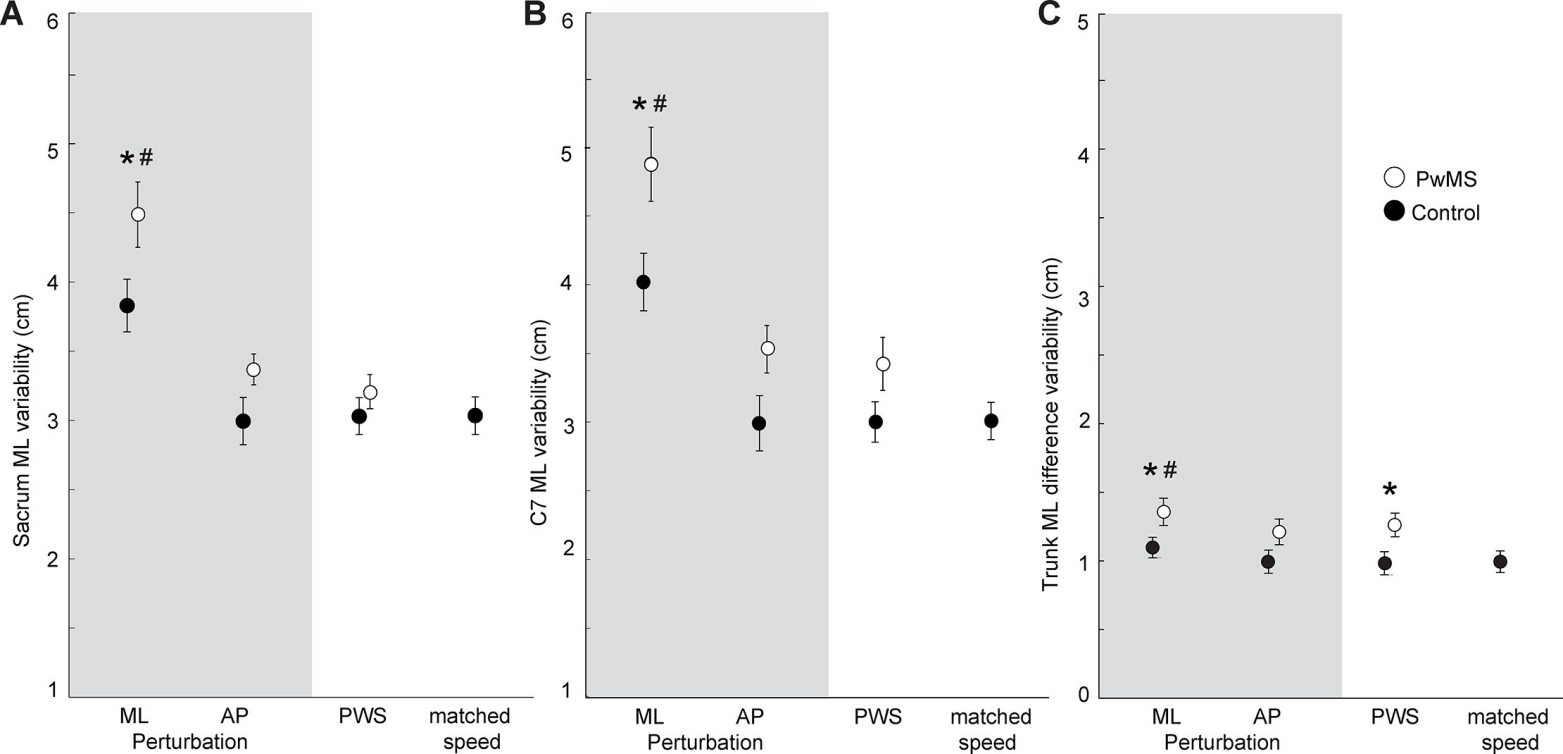
Postural outcomes in PwMS versus age-matched controls across walking tasks. Group average (standard error) mediolateral variability of the sacrum (Panel A), seventh cervical vertebra (Panel B) position, and of the relative difference between sacrum and C7 positions (Panel C) for people with multiple sclerosis (PwMS) and age-matched controls over 1 minute of walking per condition. Conditions include mediolateral (ML) and anterioposterior (AP) optical flow perturbations and two control trials. The PWS and matched speed trials have unperturbed optical flow at the participant’s preferred walking speed and the preferred walking speed of the matched PwMS, respectively. Single asterisks (*) denote significant differences (p<0.05) between PwMS and controls for a given condition. Pound signs (#) denote significant effects (p<0.05) of perturbations compared to unperturbed walking (PWS) across both groups.

**Fig 3:**
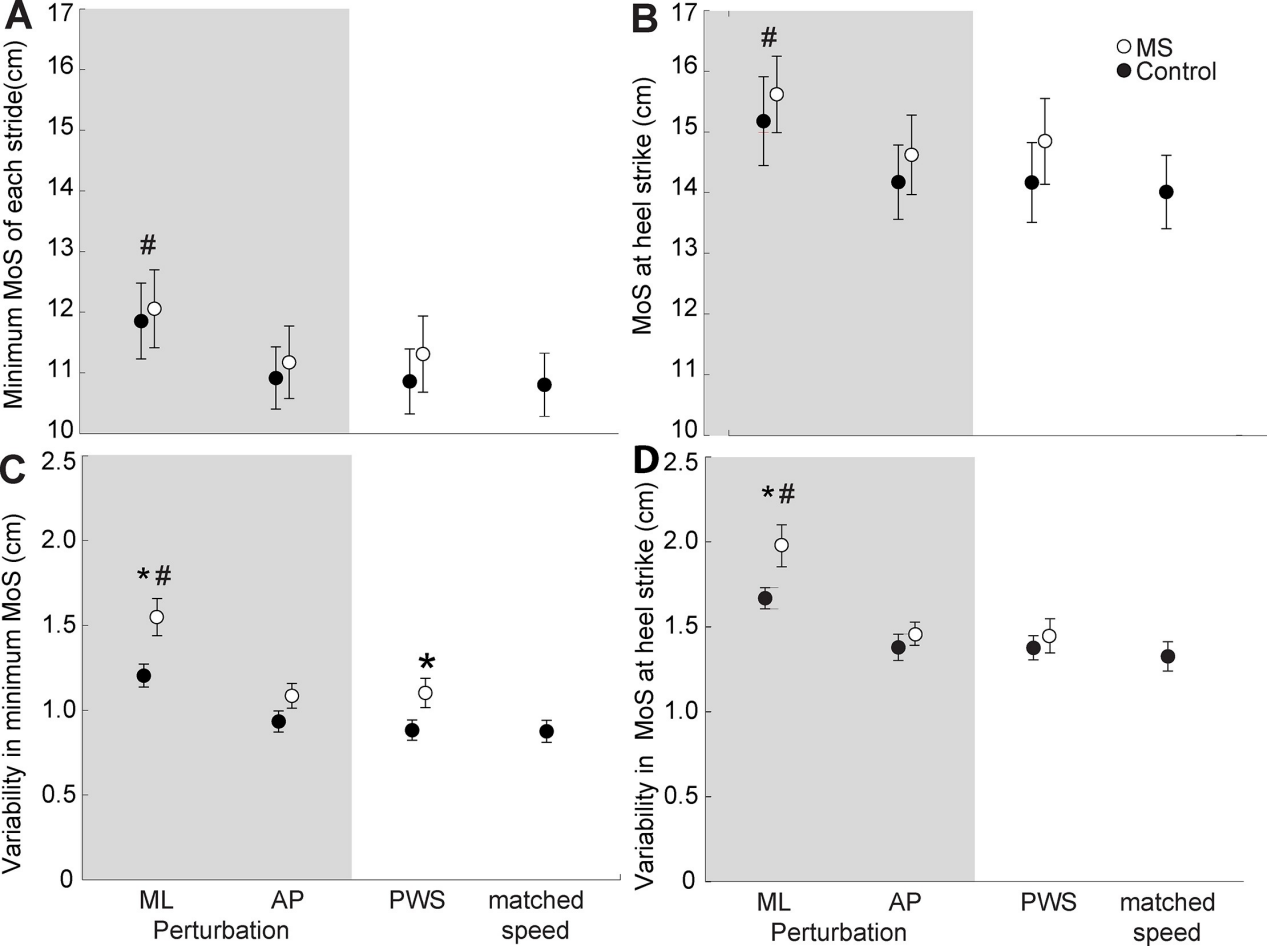
Margin of stability outcomes in PwMS versus age-matched controls across walking tasks. Group average (standard error) minimum lateral margin of stability (MoS) per stride (Panel A), lateral MoS at heel-strike (Panel B) and within subject variability in minimum lateral MoS at minimum MoS (Panel C) and at heel-strike (Panel D) for people with multiple sclerosis (PwMS) and age-matched controls. Single asterisk (*) denote significant differences (p<0.05) between PwMS and controls for a given condition. Pound signs (#) denote significant effects (p<0.05) of perturbations compared to unperturbed walking (PWS) across both groups. ML: mediolateral; AP: anterior-posterior.

We found no significant between-group differences in step width, step length, their respective variabilities (SWV and SLV), nor in ML CoM or C7 variability (Figs 1 and 2) during normal, unperturbed walking. Of those tested, only two outcomes differed significantly between groups during unperturbed walking; compared to age-matched controls, ML trunk sway variability averaged 28.6% higher (p = 0.029, d = 0.87, Fig 2) and minimum MoS variability averaged 25.0% higher (p = 0.046, d = 0.80, Fig 3) in PwMS.

### Effects of optical flow perturbations in PwMS compared to controls

We found significant main effects of condition on step width, step length, SWV, SLV, and ML CoM and C7 variability (p<0.001 for all). Post-hoc comparisons revealed that these effects–namely, wider and shorter steps and higher variability–were driven in all cases by differences exclusive to ML perturbations compared to unperturbed walking (Fig 2, p<0.001 for all). Moreover, although we found no significant condition x group interactions, ML perturbations revealed between-group differences in several outcome measures that were not apparent during unperturbed walking. Specifically, only in the presence of ML perturbations, we found that PwMS adopted 29.1% higher SWV (p = 0.022, d = 0.94; p = 0.040, Fig 1), 17.2% higher ML CoM variability ((p = 0.040, d = 0.82), and 21.4% higher ML C7 variability (p = 0.019, d = 0.95) than their age-matched controls (Fig 2). These effects were unrelated to between-group differences in walking speed; only step length differed significantly between walking at preferred speeds versus matched speeds (p = 0.008).

**Fig 4:**
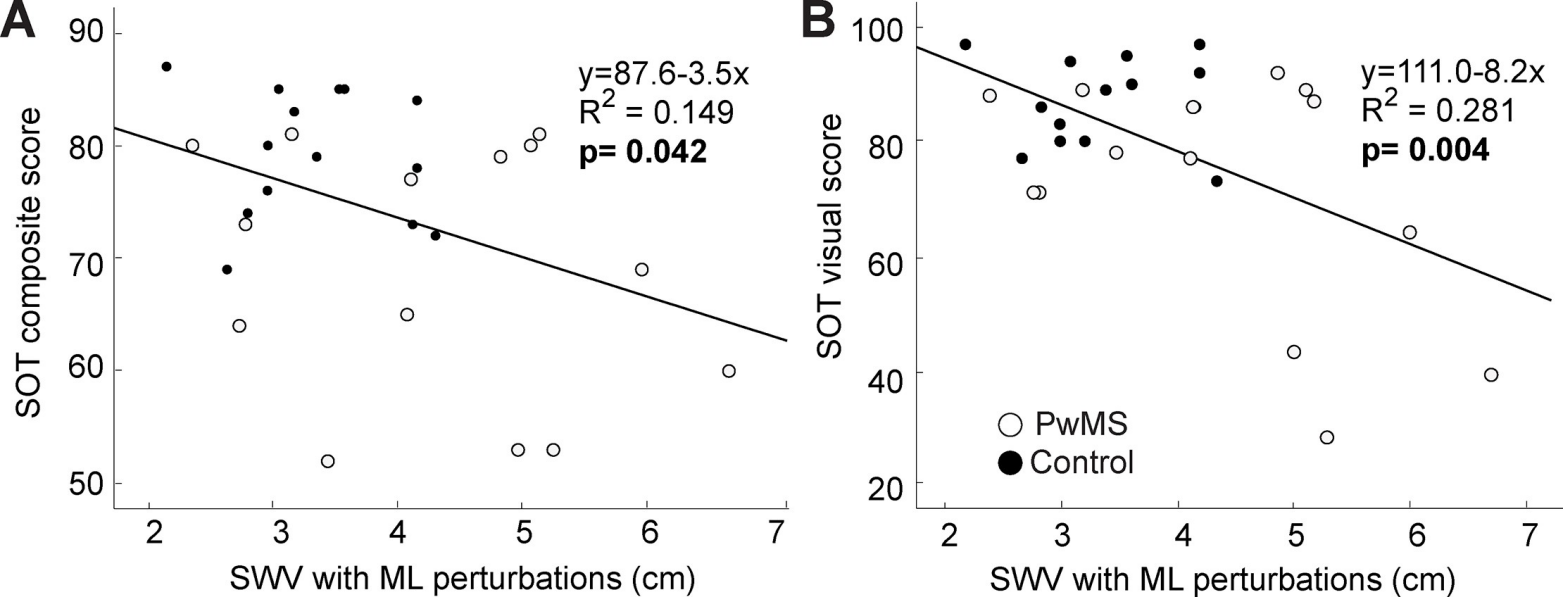
Correlations between sensory performance scores and step width variability. Correlations between sensory organization test (SOT) scores and step width variability (SWV) when walking with mediolateral optical flow perturbations, pooled across PwMS (open symbols) and age-matched controls (closed symbols). The composite score (Panel A) and visual sub-score (panel B) had significant negative correlations with SWV. We did not find a statistically significant correlation between vestibular (R^2^ = 0.119, p = 0.073) or somatosensory (R^2^<0.001, p = 0.964) sub-scores and SWV (not pictured).

Fig 3 summarizes group and condition effects on stride-averaged and step-to-step variability of lateral MoS outcomes. We found significant main effects of condition on minimum MoS, MoS at heel-strike, and their respective variabilities (p<0.001 for all). Like those for foot placement, post-hoc comparisons revealed that these effects were driven by differences exclusive to ML perturbations compared to unperturbed walking. For example, compared to unperturbed walking, minimum MoS and MoS at heel-strike averaged 7.8% and 6.1% larger for ML perturbations, respectively (p<0.001 for both, Figs 3A and 4B). However, we found no between-group differences in stride-averaged MoS for any condition. Conversely, ML perturbations revealed between-group differences in MoS variability at heel-strike that were not apparent during unperturbed walking. Specifically, only in the presence of ML perturbations, PwMS walked with 18.5% higher variability in MoS at heel-strike than their age-matched controls (p = 0.039, d = 0.84). Similarly, PwMS walked with 28.9% higher variability in minimum MoS than their controls in the presence of ML perturbations (p = 0.013, d = 1.02), a between-group effect that was 57% larger in the presence of ML perturbations than during unperturbed walking.

### Correlations between sensory performance scores and step width variability

During walking with ML perturbations, SWV tended to be larger in subjects with lower SOT scores. We found that SWV in the presence of ML perturbations exhibited a modest but statistically significant negative correlation with SOT composite score (p = 0.042, R^2^ = 0.149, Fig 4). This relation was most explained by deficits in the SOT visual score, for which we found the strongest and most significant negative correlation with SWV during ML perturbations (p = 0.004, R^2^ = 0.281).

## Discussion

This pilot study investigated the effects of optical flow perturbations on foot placement kinematics, gait variability, and lateral MoS during walking in PwMS with minimal walking impairment compared to age-matched controls. We found only modest differences between groups during normal, unperturbed walking. These differences were larger and more pervasive in the presence of ML perturbations, and, in PwMS, included significantly higher SWV, ML sacrum variability, and MoS variability at heel-strike compared to age-matched controls. These findings support our first hypothesis that, compared to age-matched controls, walking balance in PwMS would be more susceptible to optical flow perturbations, particularly to those in the ML direction. We interpret these findings to suggest that optical flow perturbations have potential as a tool for detecting preclinical walking balance impairment in PwMS that may go otherwise undetected.

During normal walking, PwMS displayed few differences from their age-matched controls. For functional context, PwMS in this study walked at slower preferred and maximum speeds, consistent with prior studies [12, 14, 45]. However, the difference in preferred speed (0.07 m/s) was modest and smaller in magnitude than the minimal clinically important difference (0.10–0.20 m/s) [46]. Of the variability outcomes analyzed, only minimum MoS variability was higher in PwMS than controls during normal walking. Although prior work indicates that PwMS have higher foot placement variability than controls during normal walking, this is generally reported for PwMS who need assistance to walk or have a history of falls [13, 44]. Indeed, SLV increases with increasing disability score in PwMS [44]. Thus, it is likely that our PwMS had not progressed to disability that would incur higher foot placement variability. Overall, foot placement kinematics and variability outcomes during unperturbed walking provide little evidence that walking balance integrity in our PwMS was worse than that of their controls. Unfortunately, even PwMS with little to no clinically measurable disability face an increased risk of falls [7, 47]. Accordingly, there is a need for more sensitive assessments of walking balance integrity for PwMS.

Optical flow perturbations revealed preclinical balance impairment in PwMS that were unapparent during normal, unperturbed walking; however, these responses were direction-dependent. Indeed, no outcome measure in either cohort was significantly influenced by optical flow perturbations designed to elicit the visual perception of speed fluctuations (i.e., AP perturbations). This finding supports our second hypothesis and is consistent with prior literature findings visual feedback is used more for controlling ML than AP balance during walking [19]. For example, using a similar optical flow paradigm, O’Connor and Kuo found that ML gait variability was much more sensitive to ML perturbations than AP gait variability was to AP perturbations [18]. During walking, this difference is likely due to relatively more passive stability in the direction of travel, which increases resilience to AP perturbations.

Conversely, a disproportionate susceptibility to ML perturbations yielded larger, more pervasive differences in gait variability between PwMS and controls, with clinical potential for detecting walking balance impairment. For example, unlike during normal walking, we found higher SWV and sacrum variability in PwMS than in age-matched controls in the presence of ML perturbations. That these two outcomes would change in parallel is not altogether surprising; during walking, step-to-step fluctuations in ML CoM motion are governed at least in part by lateral foot placement [20, 48]. This increased variability subsequently propagates to and influences translation of the whole trunk, evidenced by higher C7 variability in PwMS than in age-matched controls. However, trunk sway variability, an outcome derived from relative motion between C7 and the body’s CoM, was not disproportionately affected in PwMS compared to age-matched controls. Together, our results suggest that susceptibility to optical flow perturbations, and thereby the between-group differences they elicited, was regulated more via step-to-step variations in foot placement than in postural control, per se.

Compared to controls, greater variability in step width and sacrum position for PwMS when walking with ML perturbations was also accompanied by variability in an important outcome derived from these constituents–namely, lateral MoS. This suggests that the relative positions of (i.e., the phase between) the ML sacrum and lateral base of support, defined as the lateral boundary of the foot, are uniquely influenced by ML perturbations during the stance phase. Although we found no between-group differences in average ML MoS for any condition, only in the presence of ML perturbations was MoS variability at heel-strike higher for PwMS than controls. MoS variability is particularly relevant to preserving walking balance and preventing falls-related injury [41]. Although this is the first study to assess step-to-step variability of MoS in PwMS, Peebles et al. reported that a smaller average ML MoS correlated with increased number of falls in PwMS [42]. For a given average MoS, higher step-to-step variability in PwMS alludes to an increased probability that any step may experience an insufficient MoS to accommodate balance disturbances during walking. Accordingly, our results promote the need for future studies that measure MoS and its variability in people with more advanced MS responding to balance perturbations during walking.

One likely explanation for the greater susceptibility of PwMS to ML perturbations is a greater reliance on visual feedback for motor planning an execution–a phenomena consistent with at least one prior study in this population. Indeed, van Emmerik et al. [49] found that standing postural stability decreased more in PwMS than controls when visual feedback was removed. A similar reliance on vision has been demonstrated in older adults, presumed to arise as a sensory response to deficits in proprioceptive and/or somatosensory acuity. Perhaps accordingly, compared to young adults with high proprioceptive acuity, older adults exhibit disproportionate increases in SWV when exposed to ML optical flow perturbations [17, 5]. Our PwMS presented with similar plantar tactile sensitivity to that of age-matched controls (Semmes-Weinstein, Table 1). However, our relatively coarse assessment of somatosensory acuity may have missed deficits in other components of peripheral sensation, such as joint position sense. PwMS are less able to rely on proprioception to maintain balance than controls, whether due poor proprioceptive acuity or poor integration of proprioceptive feedback [3, 50]. Furthermore, vestibulopathy has been reported in 86% of those with relapsing-remitting MS [51]. Ultimately, proprioceptive and/or vestibular deficits in PwMS could trigger a cascade toward sensory reweighting of balance control toward visual feedback and thereby an increased susceptibility of gait variability to ML optical flow perturbations. We also posit that optical flow perturbations, by design, elicit a visual of instability that would not otherwise be possible or practical, for example via the removal of visual feedback via closing one’s eyes. However, continued study is warranted to optimize the application of this paradigm to promote clinical feasibility.

SOT scores were generally consistent with prior studies of PwMS. The visual and vestibular scores in PwMS were similar to those found by Doty and colleagues [52]. Unlike their cohort, our PwMS did not have significantly lower somatosensory scores than their controls. The condition used to determine somatosensory contribution to balance control requires standing with eyes closed but no movement of the platform, making it the easiest non-baseline condition of the SOT. The somatosensory scores observed in out PwMS– 96.6/100, only 1.3 points lower than controls–speak to the largely pre-clinical nature of their balance deficits. Indeed, Hebert et al. reported that PwMS deemed to have a fit into one lower and one higher ability group based on their SOT performance achieved [6]. The higher ability group had composite scores similar to those that participated in our study. Visual SOT scores most strongly correlated with SWV when walking with ML perturbations. This suggests that individuals with evidence of poor perceptual motor coordination–specifically, visuomotor dysfunction–in the context of standing balance were also more susceptible to optical flow perturbations during walking. Nevertheless, even this relation was modest, which may allude to the need for walking-specific tests to assess the relation between sensory acuity and/or integration and measures of walking balance.

Aside from SOT scores, between-group differences in demographic, clinical balance tests and survey data were non-significant or modest, indicating that PwMS in this study had relatively high levels of physical function compared to those in other studies. Compared to their controls, PwMS had similar BBS scores and confidence in their balance, and their higher perceived fatigue was driven more by cognitive than by physical fatigue. Thus, higher gait variability in the presence of perturbations in our PwMS is potentially indicative of underlying pre-clinical balance impairment despite their relatively high level of physical function.

Our sample size may be considered relatively small, particularly given the heterogeneity common among PwMS. As the sensory influence of central lesions associated with MS can vary across the population, different PwMS may have different levels of dependence on vision. However, our study produced between-group post-hoc statistical powers of 0.78, 0.68 and 0.79 for SWV, ML COM variability, and ML C7 variability, respectively. Given the diverse presentations of MS in patients, our results may have limited external validity to the entire population of those with MS. We therefore encourage future studies that better assess the translational relevance of susceptibility to optical flow perturbations in the diagnosis of balance impairment in PwMS. For example, a cohort spanning a broader range of disease progression, and thus physical disability and falls risk, could better associate the outcomes in this study to conventional clinical outcomes.

The optical flow portion of this study has several other limitations. Participants walked at their preferred speed, which were faster for controls. However, differences in gait speed were small, and, during unperturbed walking, speed alone did not affect variables relevant to our hypotheses. Additionally, participants were evaluated using treadmill walking, which may elicit a more cautious gait pattern with smaller speed fluctuations [53]. Although optical flow somewhat alleviates this cautious pattern [53], participants were still limited in how much they could decelerate in response to perturbations. Indeed, individuals may normally prefer to walk more slowly to accommodate balance perturbations. Additionally, we chose constant perturbation frequencies of 0.125, 0.25 and 0.442 Hz to be consistent with optical flow perturbations that altered gait variability in prior studies [17], but entrainment to perturbation frequency is affected by the participant’s stride frequency, which varies between participants [54]. Future work should investigate how differences in stride frequency affect susceptibility to optical flow perturbations. Although participants did not acclimate to the presence of optical flow per se, we randomized the order of walking trials such that any adjustments to optical flow would not affect average differences in our primary outcomes due to the onset of perturbations. Indeed, we would expect unperturbed optical flow to have little effect on gait biomechanics, as was shown with step width in older adults [55]. We did not administer a formalized questionnaire to assess the prevalence of motion sickness in our subject cohorts. However, no subjects self-reported any discomfort while completing the protocol. Lastly, a limitation of translating the current experimental paradigm is the potential difficulty of incorporating projection-based virtual reality in the clinic. However, the optical flow perturbations in this study can be readily implemented with inexpensive and commercially available virtual reality. Future feasibility studies are most certainly warranted.

This study was also limited in the number of clinical balance tests we performed. When compared to the BBS, our results suggest that optical flow perturbations would be a more sensitive way to detect preclinical balance deficits in PwMS. However, although the BBS correlates with other tests of balance used in PwMS, it may be subject to ceiling effects. Therefore, future studies should compare gait variability responses to optical flow perturbations with other balance assessments such as the functional gait assessment, a modification of the dynamic gait index designed to avoid ceiling affects [56]. Finally, this was a pilot study of only 14 relatively young PwMS whose preferred walking speeds suggest at most mild disability. Despite the small sample size, the study produced adequate powers of 0.78, 0.68 and 0.79 when comparing PwMS to controls in SWV, ML CoM variability and ML C7 variability, respectively. Although the results are promising, given the diverse presentations of MS in patients, our results may have limited external validity to the entire population of those with MS. We therefore encourage future studies that better assess the translational relevance of susceptibility to optical flow perturbations in the diagnosis of balance impairment in PwMS. For example, a cohort spanning a broader range of disease progression, and thus physical disability and falls risk, could better associate the outcomes in this study to conventional clinical outcomes.

## Conclusions

ML optical flow perturbations revealed differences in walking balance control in PwMS that went undetected during normal, unperturbed walking. Only in the presence of ML perturbations, PwMS had higher variability in foot placement, trunk motion, and MoS at heel-strike than their age-matched controls. We suspect these differences may be explained by an increase reliance on visual feedback for walking balance control. Targeting this reliance through optical flow perturbations in the safety of virtual reality may be a promising approach to more effectively detect preclinical walking balance deficits in PwMS. These promising results indicate the need for larger studies assessing PwMS with a wider range of ages, disease progressions, and balance impairments, with the additional possibilities to incorporate similar perturbations in training paradigms to improve walking balance integrity.

## Supporting information

S1 Table Individual subject demographics and data supporting all outcomes reported in this manuscript. (XLSX)

## Abbreviations

ABC: activity-specific balance confidence scale
AP: anterior-posterior
CoM: center of mass
MoS: Margin of stability
ML: mediolateral
MFIS: modified fatigue impact scale
MS: Multiple sclerosis
PWS: preferred walking speed
SWV: step width variability
SLV: step length variability
